# Structure and Thermal Properties of 2,2′-Azobis(1*H*-Imidazole-4,5-Dicarbonitrile)—A Promising Starting Material for a Novel Group of Energetic Compounds

**DOI:** 10.3390/molecules25020314

**Published:** 2020-01-13

**Authors:** Rafał Lewczuk, Maria Książek, Katarzyna Gańczyk-Specjalska, Katarzyna Cieślak

**Affiliations:** 1 Department of High-Energetic Materials, Łukasiewicz Research Network–Institute of Industrial Organic Chemistry, Annopol 6, 03-236 Warsaw, Poland; specjalska@ipo.waw.pl; 2 Institute of Physics, University of Silesia, 75 Pułku Piechoty 1, 41-500 Chorzów, Poland; maria.ksiazek@us.edu.pl; 3 Division of High Energetic Materials, Warsaw University of Technology, Noakowskiego 3, 00-664 Warsaw, Poland; kcieslak@ch.pw.edu.pl

**Keywords:** synthesis, high-nitrogen materials, thermal analysis, azo compounds

## Abstract

A high-nitrogen compound, 2,2′-azobis(1*H*-imidazole-4,5-dicarbonitrile) (TCAD), was synthesized from commercially available 2-amino-1*H*-imidazole-4,5-dicarbonitrile. It was characterized with infrared and nuclear magnetic resonance spectroscopy. Its structure was determined by single crystal X-ray diffraction. The crystal of TCAD tetrahydrate is monoclinic, with space group P2_1_/c with crystal parameters of a = 10.2935(2) Å, b = 7.36760(10) Å, c = 20.1447(4) Å, V = 1500.27(5) Å^3^, Z = 4, and F(000) = 688. Computational methods were used in order to fully optimize the molecular structure, calculate the electrostatic potential of an isolated molecule, and to compute thermodynamic parameters. TCAD has very high thermal stability with temperature of decomposition at 369 °C. Kinetics of thermal decomposition of this compound were studied and apparent energy of activation as well as the maximum safe temperature of technological process were determined.

## 1. Introduction

**Figure 1:**
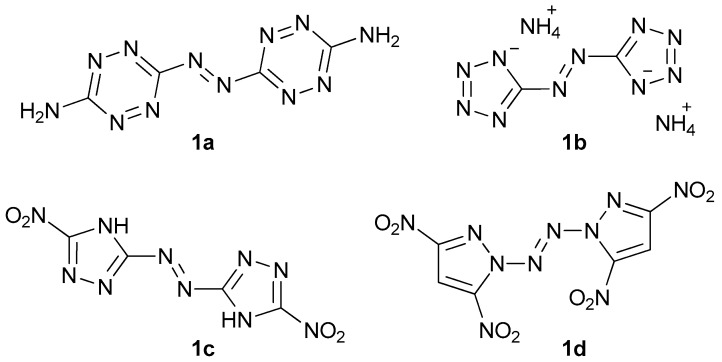
Some of the energetic azo compounds: 3,3′-azobis(6-amino-1,2,4,5-tetrazine) (**1a**), diammonium 5,5′-azobis(tetrazolate) (**1b**), 5,5′-dinitro-3,3′-azobis(1*H*-1,2,4-triazole) (**1c**), and 1,1’-azobis(3,5-dinitropyrazole) (**1d**).

Energetic materials are chemical entities with energy stored in their molecules. They belong to the various classes of organic and inorganic compounds. To name some of them, there are aliphatic and aromatic nitro compounds (nitromethane, 2,4,6-trinitrotoluene), nitroamines (ethylenedinitramine), esters (glyceryl trinitrate), and inorganic salts (lead azide, ammonium nitrate). In the last decades, we can observe an intense development of another class—aromatic heterocyclic energetics, which exhibit interesting properties suitable for many applications (e.g.: gas generants, environmentally friendly pyrotechnics) [1,2,3,4,5,6]. A distinctive group is formed by azo-bridged azoles and azines [7]. These compounds have large positive heats of formation and often show remarkable insensitivity toward electrostatic discharge, friction, and impact. Moreover, these molecules usually have better performance than parent non-linked heterocycles since they contain the azo explosophore moiety [7]. Amongst the most important energetic azo compounds, there are derivatives of 1,2,3,4-tetrazine (Figure 1, **1a**), tetrazole (**1b**), 1,2,4-triazole (**1c**), and pyrazole (**1d**) [8,9,10,11]. Nitrated imidazoles are of great interest due to their low sensitivity towards various stimuli and high detonation parameters, but no compounds with 2,2′-azobis(1*H*-imidazole) as a core have been synthesized with their possible explosive properties in mind [12,13,14,15].

**Figure 2:**
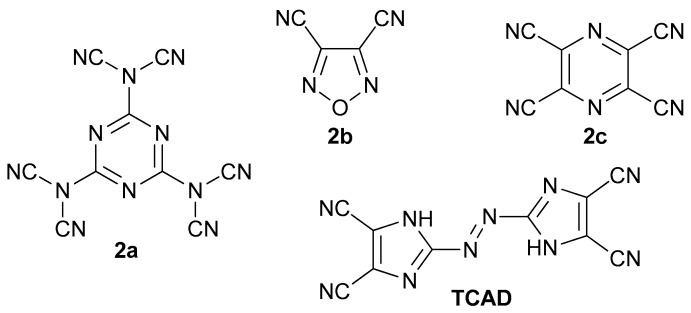
Structures of known energetic polynitriles.

Nevertheless, several derivatives with aliphatic or aromatic substituents have been reported and they may serve as photoswitches and dyes [16,17,18,19,20,21]. 2,2′-Azobis(1*H*-imidazole-4,5-dicarbonitrile) (TCAD), its alkyl derivatives, salts, metal complexes, hydrolysis products, hydrazine, and hydroxylamine adducts were recognized as dyes as well [22]. Taking into consideration that some of the polynitriles, such as *N*,*N*’,*N*”-(1,3,5-triazine-2,4,6-triyl)tris(*N*-cyanocyanamide) (Figure 2, **2a**), 1,2,5-oxadiazole-3,4-dicarbonitrile (**2b**), and pyrazine-2,3,5,6-tetracarbonitrile (**2c**), were recognized to be energetics and substrates for high-nitrogen compounds, TCAD with its azo bridge is likely to be even more energetic [23,24,25].

Therefore, for the first time, we present the molecular structure of TCAD in the crystalline state, determined using low-temperature single crystal X-ray diffraction. TCAD crystallizes from water with four water molecules per TCAD molecule in the monoclinic space group *P*2_1_/*c*, with a density of 1.480 g cm^−3^ at 100 K and four molecules in the unit cell. The geometry of the molecule was optimized using the B3LYP/cc-pVDZ level of theory. Afterwards, the enthalpy of formation of TCAD was calculated using CBS-4M with the Gaussian 09 (Revision E.01) software [26]. The prepared compound was characterized by elemental analysis, nuclear magnetic resonance, and infrared spectroscopy. It has exceptionally high decomposition temperature so herein the results of the studies of the kinetics of thermal decomposition are reported.

## 2. Results and Discussion

### 2.1. Crystal Structure

**Figure 3:**
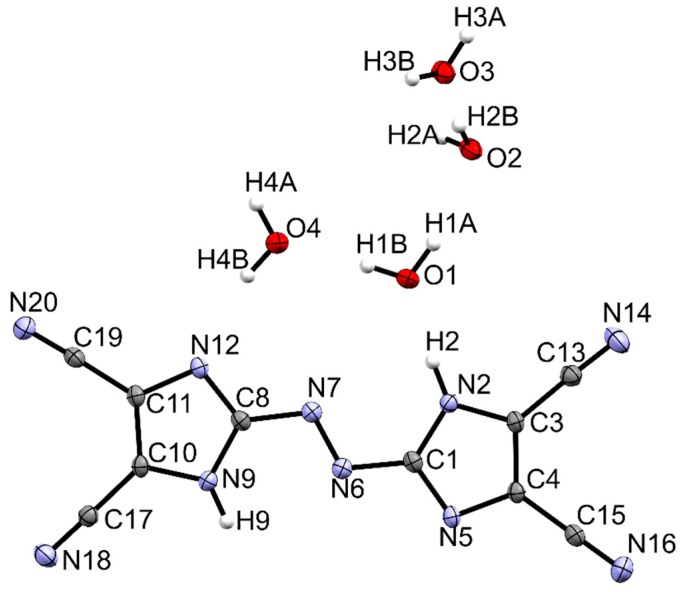
Crystal structure of TCAD·4H_2_O. Ellipsoids are scaled at 50% probability.

The title compound crystallizes in the monoclinic *P*2_1_/*c* space group with one molecule of TCAD and four water molecules. Imidazole rings lie in a plane which suggests that bonds are strongly coupled. The C8-N7 bond length is 1.402 Å and the length of the N=N bond of the azo bridge is 1.266 Å. Similar angles and distances can be found in other azo derivatives of azoles: 4,4′-azobis(3,5-diazido-1,24-triazole) (1.38 Å, 1.25 Å, resp.) [27], 3,3′-azobis(5-(trinitromethyl)-1,2,4-triazole) (1.39 Å, 1.24 Å, resp.) [28], and in diammonium 5,5’-azobis(tetrazolate) (1.40 Å, 1.26 Å, resp.) [29]. All mentioned azo-bridged molecules are planar. The molecular structure of TCAD tetrahydrate and atomic numbering were shown in Figure 3. In Table 1, the crystal parameters, experimental, and refinement details are collected.

Crystal structure exhibits strong hydrogen bonding. Hydrogen bonds are formed between the N–H as H-bond donors (D) and N or O atoms as H-bond acceptors (A). The presence of water molecules in the crystal structure also promotes the formation of hydrogen bonds. There can be distinguished two types of such bonds—between water molecules and the molecule of TCAD (O–H···N) and only between water molecules (O–H···O). According to the classification proposed by Desiraju and Steiner, the O–H···O and N–H···O interactions are strong, while the N–H···N and O–H···N hydrogen bonds are weak [30]. H-bond parameters for the studied compound and the results of the Hirshfeld surfaces analysis were collected in the Supporting Information.

### 2.2. Physiochemical Properties

The standard molar enthalpy of formation and standard molar enthalpy of combustion of TCAD are equal to 960 kJ mol^−1^ (3661 kJ kg^−1^) and −5181 kJ mol^−1^ (−19.76 kJ·g^−1^), respectively. The relatively high enthalpy of formation can be attributed to the azo-bridged (Δ*_f_*H°_(g)_: *trans*-HN=NH = 201.3, *trans*-H_3_C-N=N-CH_3_ = 147.7 *versus* H_3_CCH_3_ = −83.8 [kJ mol^−1^]) imidazoles skeleton (Δ*_f_*H°_(s)_: 1*H*-imidazole = 49.8, 1*H*-pyrazole = 105.4 [kJ mol^−1^]) [31,32,33]. Further functionalization of the imidazoles rings in 4, 5 positions via [2 + 3] dipolar azide-nitrile cycloaddition reaction could yield in a highly promising energetic material, namely the tetratetrazolo-congener (N = 70.95%).

**Figure 4:**
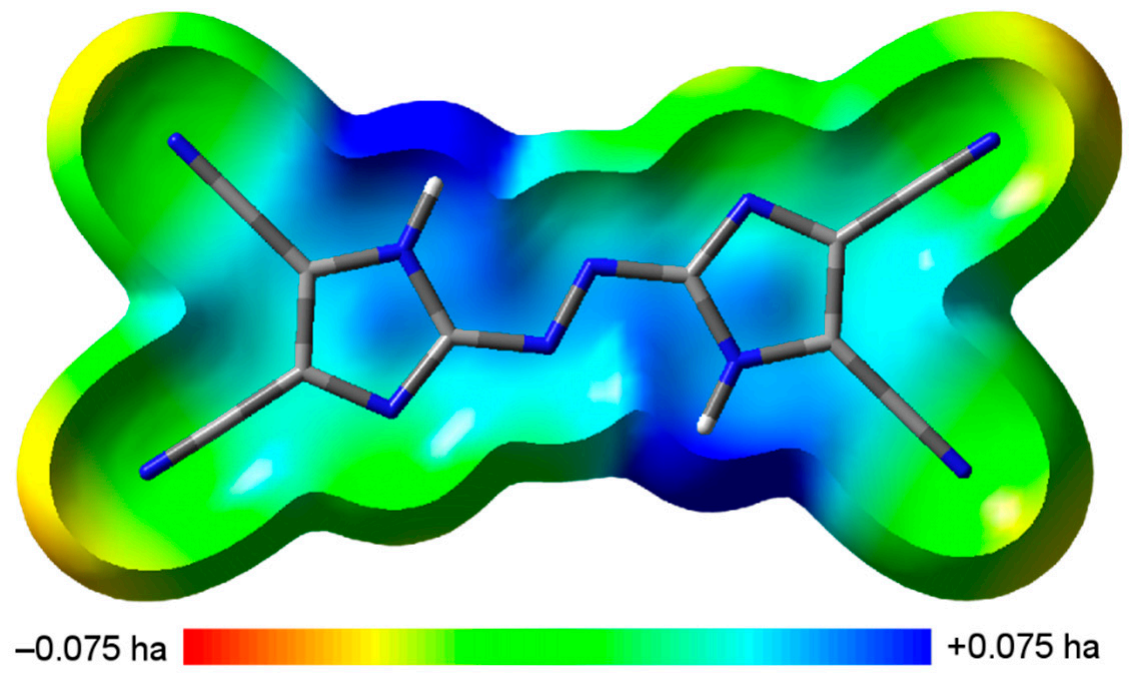
Calculated electrostatic potential of TCAD (view perpendicular to the plane).

The sensitivity to impact of a compound can be connected to the electrostatic potential (ESP) of an isolated molecule [34,35,36,37]. The ESP of TCAD on the 0.001 electron bohr^−3^ hypersurface, with the color coding ranged from red (*V*(*r*) ≤ −0.075 hartree) for electron-rich regions to blue (*V*(*r*) ≥ +0.075 hartree) for electron-deficient regions, is shown in Figure 4 [38].

TCAD can be characterized by stronger and much smaller negative electrostatic potential regions than positive ones, which can be attributed to low-impact-sensitive covalent species (details are given in the Supporting Information) [34,35,36,37]. Sensitivity to mechanical stimuli of anhydrous TCAD was also determined experimentally. There was no positive response for the impact energy 30 *J* and for the friction force 360 *N*. It indicates insensitivity of the tested compound. Experimental results are in accordance with computations.

During differential thermal analysis (DTA) coupled with thermogravimetry (TG) measurement with a heating rate β = 5 °C min^−1^, mass loss of TCAD proceeded in two steps. The first one began at 118 °C (−1.7%), the second one at 340 °C (−23.4%). First, the endothermic signal was observed on a DTA curve at 147.1 °C (maximum peak temperature) and it can be assigned to the loss of water which was not removed during the drying process. Second, the signal at 370.2 °C was exothermic and it was connected to thermal decomposition. The temperature of decomposition, the onset point of the peak, was 368.7 °C.

**Figure 5:**
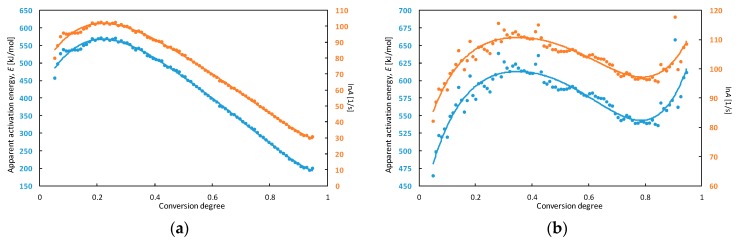
The dependence of apparent activation energy (*E*) and the natural logarithm of the pre-exponential factor (ln*A*) on the conversion degree for TCAD achieved in DTA (**a**) and TG (**b**) measurements.

As expected, the decomposition process had occurred at a higher temperature, as the heating rate was increased. The maximum peak temperatures were observed in a range from 354 °C to 374 °C. Another exothermic process was observed on the right sides of peaks for some heating rates (5 and 10 °C min^−1^). This means that decomposition of TCAD is a complex process—multiple reactions may take place simultaneously and/or sequentially during transition. Because of this reason, the calculated activation energy in the next part of this manuscript was called apparent activation energy. The kinetic analysis of the decomposition process was determined using DTA and TG curves. The dependences of apparent activation energy (*E*) and the natural logarithm of the pre-exponential factor (ln*A*) on the conversion degree for TCAD achieved in DTA and TG measurements are shown in Figure 5.

The characteristics of ln*A* and *E* change with a similar pattern. The apparent activation energy calculated from results of DTA experiments increased from approx. 480 to 570 kJ mol^−1^ and ln*A* increased from approx. 85 to 103 in a range of conversion degree α = 0.05–0.20. Then, *E* decreased to 200 kJ mol^−1^ and ln*A* to 30.

The dependences calculated from TG measurements correspond to three-stage processes. In the range of conversion degree α = 0.05–0.35, apparent activation energy increased from approx. 480 to 610 kJ mol^−1^ then decreased to 540 kJ mol^−1^ (α = 0.80) and increased again to 615 kJ mol^−1^ (α = 0.95). In the first stage, ln*A* increased from 85 to 110. Next, it decreased to 97 (α = 0.80) and increased to 110 (α = 0.95).

Major changes of the apparent activation energy with the conversion degree are connected with many thermally initiated process (e.g. reactions, physical transitions) taking place during TCAD decomposition. The apparent activation energy for low conversion degree (α = 0.05) equals to approx. 480 kJ mol^−1^ for both DTA and TG measurements. Since the runaway decomposition of high-energetic materials can occur for low conversion degree and it can lead to thermal explosion, that value could be used for determination of thermal stability of the compound [39,40]. The parameters which allows to analyze the thermal explosion hazard are e.g., maximum safe operating temperature of technological processes and self-accelerating decomposition temperature [41]. The 100 °C rule is the simplest method for determination of maximum safe temperature of technological process based on DTA measurements [42]. The maximum safe temperature is 100 °C, lower than the maximum temperature of the decomposition peak on the DTA curve (measurements for 10 °C min^−1^). The maximum temperature of TCAD decomposition is 373.5 ± 0.7 °C and the maximum safe temperature of technological process for this compound equals to 274 °C, according to the 100 °C rule.

## 3. Materials and Methods

### 3.1. General Information

High-purity sodium nitrate(III), sodium sulphate(IV), and hydrochloric acid were purchased from Sigma-Aldrich (Steinheim, Germany). 2-Amino-4,5-dicyano-1*H*-imidazole (ABCR, product no. AB136180) was used as supplied. Melting point of the product was determined with an Electrothermal 9200 melting point apparatus. Elemental analysis of TCAD was performed with a Vario El Cube CHNS-O-Cl analyzer in the CHN mode. The Fourier transform infrared spectroscopy (FTIR) analysis was performed using Thermo Scientific NICOLET 6700 spectrometer (Waltham, MA, USA) with ATR (Attenuated Total Reflectance) unit. The FTIR spectra was recorded in the wave number range: 400–4000 cm^−1^ with a resolution of 2 cm^−1^. ^1^H and ^13^C-NMR spectra were recorded on a Bruker Avance III spectrometer (Bruker, Coventry, UK) (500 and 125 MHz, respectively). The chemical shifts in ^1^H and ^13^C-NMR spectra were given with respect to TMS as the external standard. The mechanical sensitivity was determined by using standard BAM fall hammer for the impact sensitivity (IS) and BAM friction tester for friction sensitivity (FS). Tests were conducted according to United Nations recommendations [43].

### 3.2. Synthesis

TCAD was obtained according to Carlson’s method [22].

2-Amino-1*H*-imidazole-4,5-dicarbonitrile (75 mmol, 9.98 g) was dissolved in water (300 mL), mixed with 38% water solution of hydrochloric acid (66 mL) at 0 °C. Afterwards, a solution of sodium nitrite (130 mmol, 8.97 g) in water (25 mL) was added dropwise over 10 minutes. The mixture was stirred for 1 h at 0 °C. Subsequently, a solution of anhydrous sodium sulfite (90 mmol, 11.34 g) in water (60 mL) was added at once to the vigorously stirred suspension of, generated in situ from obtained 4, 5-dicyanoimidazole-2-diazonium chloride, 2-diazo-4,5-dicyanoimidazole [44]. The bright yellow mixture was stirred for 30 min at 0 °C and filtered. The yellow precipitate was washed with acidified cold water and dried at 60 °C. The crude product was recrystallized from boiling water and dried at 60 °C to remove water molecules. Total yield: 2.46 g (25%). M.p. = 369–371 °C (decomp.). FTIR (cm^−1^) ν: 3162 (s), 3070 (w), 2945 (w), 2247 (vs), 1540 (w), 1490 (w), 1421 (m), 1209 (s), 1033 (s), 729 (vs); ^1^H-NMR (DMSO-*d*_6_, 500 MHz): δ 8.56 (s, 2H); ^13^C-NMR (DMSO-*d*_6_, 125 MHz): δ 112.9 (s), 119.2 (s), 159.7 (d); anal. calcd for C_10_H_2_N_10_: C, 45.81; H, 0.77; N, 53.42; found: C, 45.60; H, 0.81; N, 53.29%.

### 3.3. X-Ray Crystallography

Suitable crystal for the measurement was obtained by slow evaporation of water solution of TCAD. Crystal data were collected using a SuperNova diffractometer (Agilent Technologies, Santa Clara, CA, USA) with Atlas detector using mirror monochromator (MoK*_α_* radiation, λ = 0.71073 Å). Accurate cell parameters were determined and refined using the CrysAlis^Pro^ program (ver. 1.171.38.41q, Rigaku Oxford Diffraction, 2015, Wrocław Poland). For the integration of the collected data, the same program was used. The structures were solved using the direct method with SHELXS-2013 software (Universität Göttingen, Lower Saxony, Germany) and the solution was refined with SHELXL-2018/3 [45]. All non-hydrogen atoms were refined with anisotropic displacement parameters. Hydrogen atoms were treated as “riding” on their parent carbon atoms with *d*(C–H) = 0.88 Å and *U*_iso_(H) = 1.2 *U*_eq_(C) for aromatic H and N–H atoms. Water molecules were refined as a rigid group with *d*(O–H) = 0.87 Å and *U*_iso_(H) = 1.5 *U*_eq_(O). The details relating to the atomic coordinates, complete geometry of molecules, and all crystallographic data are deposited at the Cambridge Crystallographic Data Center (CCDC). CCDC-1862760 contains the supplementary crystallographic data for this paper. These data can be obtained free of charge via http://www.ccdc.cam.ac.uk/conts/retrieving.html.

### 3.4. Computations

**Scheme 1:**
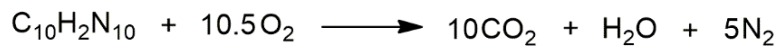
The chemical equation for stoichiometric combustion of TCAD in oxygen.

The structure of TCAD was fully optimized without any symmetry constraints and was found to represent true minima (*C_2h_* symmetry; number of imaginary frequencies, NIMAG = 0). The gas phase absolute molar enthalpy of TCAD at the standard conditions (298 K and 1 atm) was computed theoretically, applying the modified complete basis set method (CBS-4M) with the Gaussian 09 (Revision E.01) software [26]. The gas phase molar enthalpy of formation at the standard conditions of TCAD was calculated using the atomization energy method [46,47,48]. The standard molar enthalpy of formation (Δ*_f_*H°_(s)_) was calculated using the gas phase standard molar enthalpy and the standard molar enthalpy of sublimation (applying Trouton’s rule) [49]. Additionally, standard molar enthalpy of combustion (Δ*_c_*H°_(s)_) was calculated assuming the reaction shown in Scheme 1 (details are given in the Supporting Information).

### 3.5. Thermal Analysis

DTA/TG analyses were performed using Setaram Labsys^TM^ Evo calorimeter (Caluire, France). All measurements were done in open aluminum crucibles in argon atmosphere. Mass of each sample was 7.5 ± 0.2 mg. DTA/TG analyses were carried out using five different heating rates from 0.5 to 10 °C –min^−1^; the measurements for minimum and maximum heating rates were repeated three times in accordance with recommendations [50]. Kinetic parameters of the decomposition process were calculated using AKTS Thermokinetics Software. The activation energy and pre-exponential factor were calculated using the differential isoconversional method.

## 4. Conclusions

2,2′-Azobis(1*H*-imidazole-4,5-dicarbonitrile) can be easily synthesized from commercially available substrates. It crystallizes from water as a tetrahydrate, of which the crystal structure was determined with single crystal X-ray crystallography. TCAD is planar and it is hydrogen-bonded with molecules of water. The calculated standard molar enthalpy of formation is very high (960 kJ mol^−1^) which is caused by significant contribution of the azo bridge. Strong negative electrostatic potential regions in the molecule indicate low sensitivity to impact. Large delocalization of electrons in the structure results in very good thermal stability. Temperature of decomposition is nearly 370 °C. The apparent activation energy for the low conversion degree was calculated from DTA and TG results and it was very high (480 kJ mol^−1^). Finally, the maximum safe temperature of the technological process for TCAD was determined at 274 °C.

Taking into consideration foregoing results, it could be concluded that TCAD is an interesting energetic molecule with excellent thermal stability and low sensitivity. It is a promising starting material for high-nitrogen compounds such as 2,2′-azobis[4,5-di(1*H*-tetrazol-5-yl)-1*H*-imidazole] (TTAZI) and 2,2′-azobis(4,7-diamino-1*H*-imidazo[4,5,d]pyridazine). Tetrazoles derived from **2a**, **2b**, and **2c** have decomposition temperatures way above 200 °C and 4,5-di(1*H*-tetrazol-5-yl)-1*H*-imidazol-2-amine decomposes at 227 °C [51], so TTAZI with its π-electron conjugations could also have very good thermal stability. TCAD itself, which is a high-nitrogen nitrile similar to dicyandiamide, has a potential to be used in pyrotechnic fire extinguishers as a coolant and reductant, which can produce large quantities of nitrogen when combusted. Furthermore, 2,2′-azobis(1*H*-imidazole) has huge potential to be a core structure in a brand new family of explosives with nitro-, nitramino-, and amino-substituents. The fact that protons in TCAD are acidic can lead to a whole group of energetic salts and complex compounds. The possibilities mentioned above endorse to continue research on 2,2′-azobis(1*H*-imidazole) derivatives.

## Figures and Tables

**Table 1:** Crystal data and structure refinement for TCAD·4H_2_O.

Parameter	Value
Empirical formula	C_10_H_10_N_10_O_4_
Formula weight [g/mol]	334.28
Temperature [K]	100(1)
Wavelength [Å]	0.71073
Crystal system	monoclinic
Space group	P2_1_/c
Unit cell dimensions:	
a [Å]	10.2935(2)
b [Å]	7.36760(10)
c [Å]	20.1447(4)
β [°]	100.882(2)
Volume [Å^3^]	1500.27(5)
Z	4
Density [g/cm^3^]	1.480
Absorption coefficient [mm^−1^]	0.119
F(000)	688
Crystal size [mm]	0.07 × 0.13 × 0.27
Theta range for data collection [°]	2.950 to 26.370
Index ranges	−12 ≤ *h* ≤ 12, −9 ≤ *k* ≤ 7, −25 ≤ *l* ≤ 25
Reflections collected	12183
Independent reflections	3067 [R_(int)_ = 0.0159]
Completeness to theta=25.242° [%]	99.9
Refinement method	Full-matr–ix least-squares on F^2^
Data/restraints/parameters	3067/0/229
Goodness-of-fit on F^2^	1.035
Final R indices [I > 2sigma(I)]	R1 = 0.0341, wR2 = 0.0921
R indices (all data)	R1 = 0.0364, wR2 = 0.0947
Largest diff. peak and hole	0.396 and –0.224

## Supplementary Materials

The following are available online, Figure S1: The fragment of the net of the inter- and intramolecular contacts in TCAD·4H_2_O, Figure S2: Two-dimensional fingerprint plot for the compound TCAD·4H_2_O, Figure S3: The 2D fingerprint plot resolved into N···H (left), H···N (center), and N···N (right) interactions for TCAD·4H_2_O, Figure S4: Calculated electrostatic potential of TCAD (view perpendicular to the plane through the atoms), Figures S5–S7: NMR spectra of TCAD, Figure S8: FT-IR spectrum of TCAD, Figure S9: DTA/TG thermogram of TCAD (heating rate: 5 °C/min), Figure S10: DTA curves of TCAD for various heating rates, Figure S11: TG curves of TCAD for various heating rates, Table S1: Selected hydrogen bond parameters in TCAD·4H_2_O, Table S2: Intermolecular contacts and their contributions to the Hirshfeld surface in TCAD·4H_2_O, Table S3: Literature values for $\Delta_{f}H_{\left(g,\;A_{i},\;298K\right)}^{{}^{\circ}}$ and $H_{\left(g,\;A_{i},\;298K\right)}^{{}^{\circ}}$ obtained from theoretical calculations at the CBS-4M level of theory, Table S4: CBS-4M, gas-phase enthalpy, and standard molar enthalpy of formation for TCAD.
