# Supplementary material for: Structure and Thermal Properties of 2,2′-Azobis(1H-Imidazole-4,5-Dicarbonitrile)—A Promising Starting Material for a Novel Group of Energetic Compounds
Source: Molecules. 2020 Jan 13;25(2):314. doi: 10.3390/molecules25020314 (PMC7024350; doi:10.3390/molecules25020314)
Supplement: Supplementary file 1 [file molecules-25-00314-s001.zip › Structure and thermal properties - supporting information.pdf]

# Supporting Information

## Structure and thermal properties of 2,2′-azobis(1*H*-imidazole-4,5-dicarbonitrile) – a promising starting material for a novel group of energetic compounds

**Rafał Lewczuk [1,*], Maria Książek [2], Katarzyna Gańczyk-Specjalska [1] and Katarzyna Cieślak [3]**

[1] Department of High-Energetic Materials, Łukasiewicz Research Network – Institute of Industrial Organic Chemistry, Annopol 6, 03-236 Warsaw, Poland; specjalska@ipo.waw.pl (K.G.-S.)
[2] Institute of Physics, University of Silesia, 75 Pułku Piechoty 1, Chorzów, 41-500, Poland; maria.ksiazek@us.edu.pl
[3] Division of High Energetic Materials, Warsaw University of Technology, Noakowskiego 3, 00-664 Warsaw, Poland; kcieslak@ch.pw.edu.pl
* Correspondence: lewczuk@ipo.waw.pl

## 1. X –ray structural studies

Table S1 shows the H-bond parameters for **TCAD·4H$_2$O**. Figure S1 depicts the hydrogen bonds found in this compound.

**Table S1.** Selected hydrogen bond parameters in **TCAD·4H$_2$O**.

| *D*—H···*A* | *D*—H (Å) | H···*A* (Å) | *D*···*A* (Å) | *D*—H···*A* (°) |
|---|---|---|---|---|
| N2—H2···N7 | 0.88 | 2.58 | 2.7083 (13) | 88.6 |
| N2—H2···O1 | 0.88 | 1.73 | 2.6028 (13) | 169.0 |
| N9—H9···N6 | 0.88 | 2.51 | 2.6525 (13) | 89.7 |
| O1—H1A···O2 | 0.87 | 2.01 | 2.8412 (12) | 159.3 |
| O1—H1B···O4 | 0.87 | 1.84 | 2.6978 (12) | 169.0 |
| O2—H2B···O3 | 0.87 | 1.76 | 2.6261 (12) | 173.8 |
| O4—H4B···N7 | 0.87 | 2.97 | 3.4075 (13) | 112.8 |
| O4—H4B···N12 | 0.87 | 2.06 | 2.9050 (13) | 164.8 |
| N9—H9···O2$^{i}$ | 0.88 | 1.84 | 2.6948 (13) | 163.4 |
| O3—H3A···N5$^{ii}$ | 0.87 | 2.04 | 2.8865 (13) | 162.6 |
| O2—H2A···N18$^{iii}$ | 0.87 | 2.22 | 3.0134 (14) | 151.5 |
| O2—H2A···N20$^{iv}$ | 0.87 | 2.89 | 3.3806 (14) | 117.2 |
| O4—H4A···N18$^{iv}$ | 0.87 | 2.29 | 3.0585 (13) | 147.1 |
| O3—H3B···N16$^{v}$ | 0.87 | 2.48 | 3.0737 (13) | 126.4 |

| | | | | |
|---|---|---|---|---|
| O3—H3A···N16$^{v}$ | 0.87 | 2.89 | 3.0737 (13) | 93.6 |
| O3—H3B···N14$^{vi}$ | 0.87 | 2.24 | 2.9964 (13) | 145.4 |
| O4—H4A···N20$^{vii}$ | 0.87 | 2.56 | 3.1110 (13) | 122.3 |
| O4—H4B···N20$^{vii}$ | 0.87 | 2.86 | 3.1110 (13) | 98.5 |

Symmetry code(s): (i) *x*, -*y*+3/2, *z*-1/2; (ii) *x*, -*y*+3/2, *z*+1/2; (iii) -*x*, -*y*+1, -*z*; (iv) *x*, -*y*+1/2, *z*+1/2; (v) -*x*+1, *y*-3/2, -*z*+1/2; (vi) *x*, *y*-1, *z*; (vii) -*x*, -*y*, -*z*.

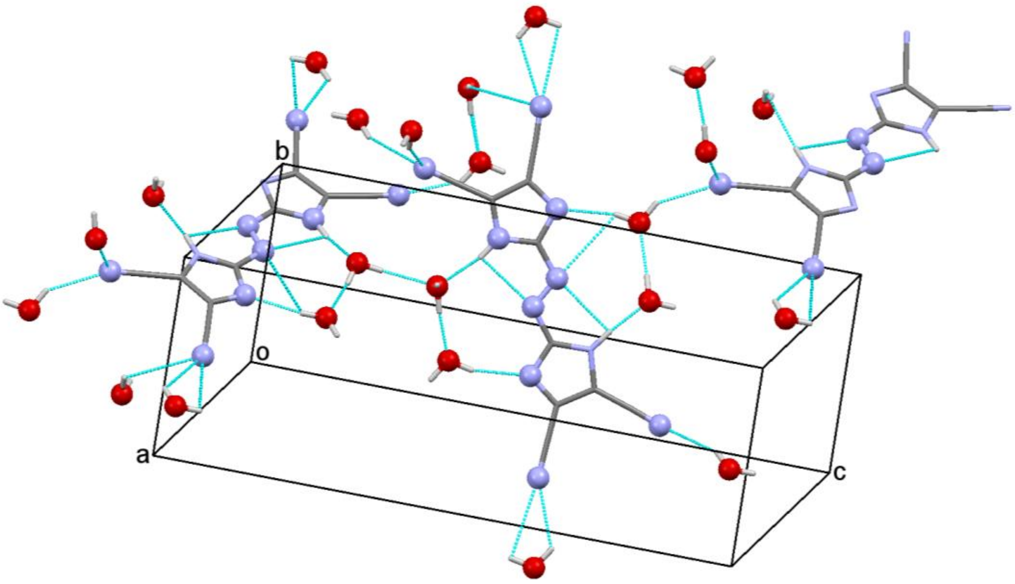


**Figure S1.** The fragment of the net of the inter- and intramolecular contacts in **TCAD·4H$_2$O.**

The Hirshfeld surfaces analysis is useful to explain the intermolecular interactions [1]. The analysis was carried out using the CrystalExplorer program [2]. The intermolecular interactions of the compound are shown in the two-dimensional (2D) fingerprint plot on Figure S2.

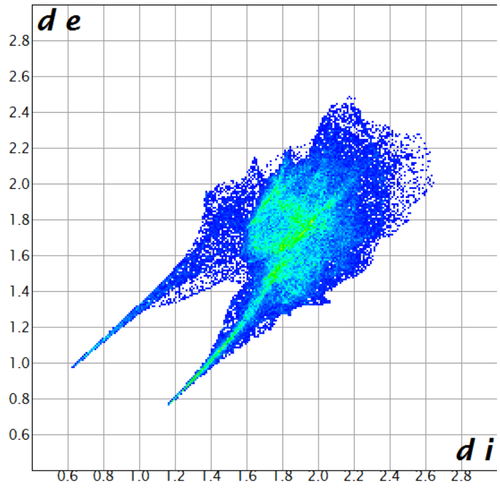


**Figure S2.** Two-dimensional fingerprint plot for the compound **TCAD·4H$_2$O.**

All interactions, which are observed in **TCAD·4H$_2$O**, are summarized in the Table S2 with their percentage contribution to the surface.

**Table S2.** Intermolecular contacts and their contributions to the Hirshfeld surface in **TCAD·4H$_2$O**.

| Type of contacts | Contribution to the surface [%] |
|---|---|
| N···N | 17.6 |
| N···H | 38.5 |
| N···C | 14.5 |
| N···O | 0.8 |
| C···H | 14.9 |
| C···C | 1.2 |
| C···O | 4.5 |
| O···H | 4.7 |
| O···O | 0.0 |
| H···H | 3.2 |

The interactions of the type N···H have the most significant contribution to the Hirshfeld surface and they are reflected in upper part and in bottom part with a spike. The area corresponding to N···H interactions is placed in the middle of the plot (Figure S3).

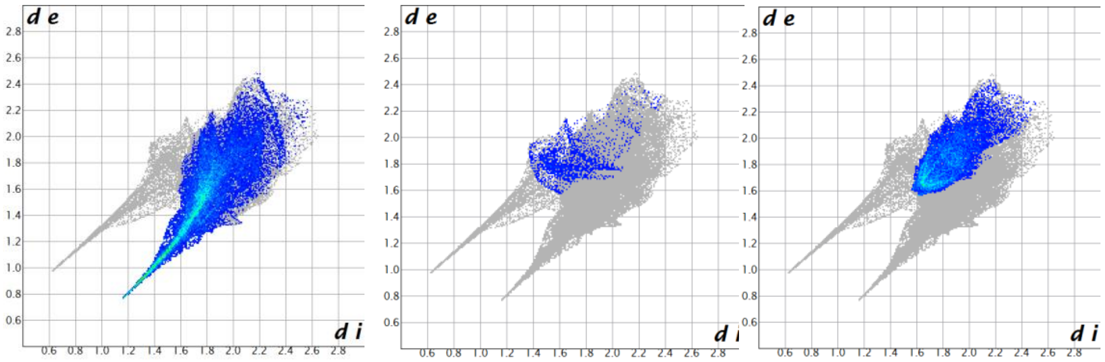


**Figure S3.** The 2D fingerprint plot resolved into N···H (left), H···N (center), and N···N (right) interactions for **TCAD·4H$_2$O**.

## 2. Calculations

### 2.1. Heat of formation

The enthalpy ($H$) was calculated using the modified complete basis set method (CBS-4M; M refers to the use of minimal population localization) with the Gaussian G09 (Revision E.01) program package [3]. The CBS-4M method is a re-parameterized version of the CBS-4 method with additional empirical corrections [4,5]. The gas-phase enthalpy ($\Delta_f H°_{(g,M,298K)}$) of the molecule $M$ was calculated applying the atomization energy method (Equation 1) [6-8].

$$\Delta_f H°_{(g,M,298K)} = H_{(g,M,298K)} - \sum H°_{(g,A_i,298K)} + \sum \Delta_f H°_{(g,A_i,298K)} \left[\frac{J}{mol}\right] \quad (1)$$

$\Delta_f H°_{(g,A_i,298K)}$ for the corresponding atoms ($A_i$) were determined experimentally whereas $H°_{(g,A_i,298K)}$ was calculated theoretically (Table S3) [9].

**Table S3.** Literature values for $\Delta_f H°_{(g,A_i,298K)}$ and $H°_{(g,A_i,298K)}$ obtained from theoretical calculations at the CBS-4M level of theory [9].

| Atom | $\Delta_f H^{\circ}_{(g,A_i,298K)}$ [kcal·mol$^{-1}$] | $H^{\circ}_{(g,A_i,298K)}$ [hartree·atom$^{-1}$] |
|---|---|---|
| H | 52.103 | −0.500991 |
| C | 171.29 | −37.786156 |
| N | 112.97 | −54.522462 |

Standard molar enthalpies of formation were calculated using $\Delta_f H°_{(g,M,298K)}$ and the standard molar enthalpies of sublimation (estimated using the Trouton's rule, Equation 2) [10].

$$\Delta_f H°_M = \Delta_f H°_{(g,M,298K)} - \Delta_{sub} H°_M = \Delta_f H°_{(g,M,298K)} - 188 \cdot T \left[\frac{J}{mol}\right] \quad (2)$$

Where $T$ is either the melting or the decomposition temperature (if melting does not occur prior to decomposition).

Results of the calculations are presented in the Table S4.

**Table S4.** CBS-4M, gas-phase enthalpy, and standard molar enthalpy of formation for **TCAD**.

| | Formula | $-H_{(g,M,298K)}$ [hartree] | $\Delta_f H°_{(g,M,298K)}$ [kJ mol$^{-1}$] | $\Delta_f H°_M$ [kJ mol$^{-1}$] |
|---|---|---|---|---|
| **TCAD** | $C_{10}H_2N_{10}$ | 928.372439 | 1081 | 960 |

## 2.2. Electrostatic Potential

The sensitivity to impact of a molecule can be connected with the electrostatic potential (*ESP*) of an isolated molecule ($V(r)$, Equation 3) [11]:

$$V(\boldsymbol{r}) = \sum_i \frac{Z_i}{|\boldsymbol{R_i} - \boldsymbol{r}|} - \int \frac{\rho(\boldsymbol{r}')}{|\boldsymbol{r}' - \boldsymbol{r}|} d\boldsymbol{r}' [Ha] \tag{3}$$

where:

$Z_i$ – the charge on nucleus $i$, placed at $R_i$ relative to $r$;

$\rho(r')$ – the electronic density at the position $r'$ relative to $r$.

The first part of the Equation 3 defines the contribution of the nuclei while the second part expresses the involvement of electrons.

The calculations of *ESP* were performed applying the B3LYP/6-311+G(2d,p) level of theory using the Gaussian G09 program package [3]. The electrostatic potential of **TCAD** on the 0.001 electron·bohr$^{-3}$ hypersurface with the colour range from red ($V(r) \leq -0.075$ Hartree, electron-rich regions) to blue ($V(r) \geq +0.075$ Hartree, electron-deficient regions) was illustrated using GaussView 5 software (Figure S4) [12].

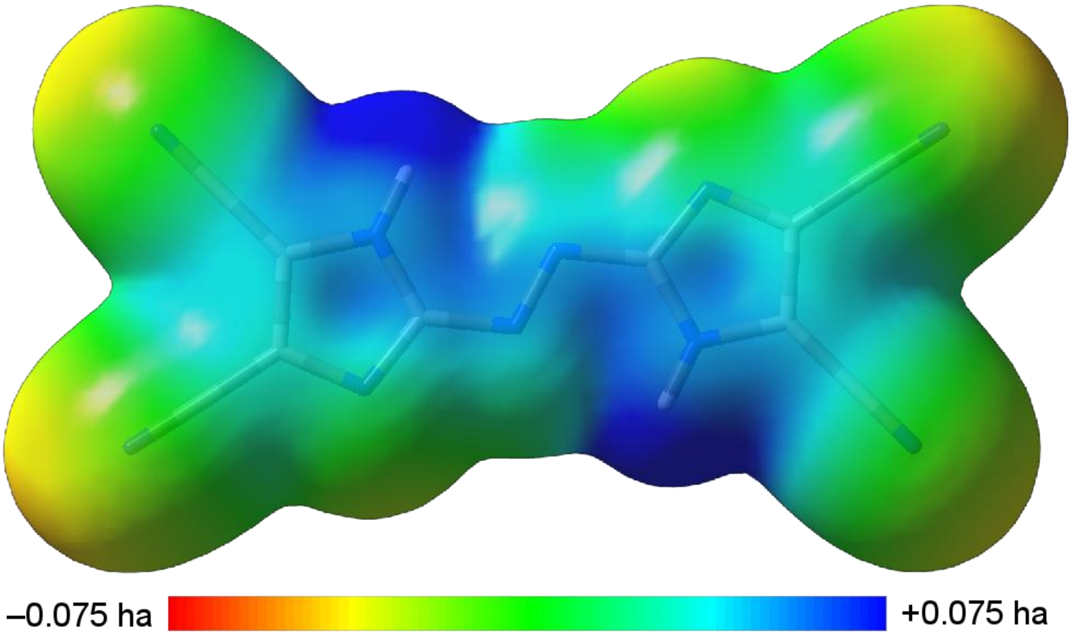


**Figure S4.** Calculated electrostatic potential of **TCAD** (view perpendicular to the plane through the atoms).

## 3. Spectroscopy

### 3.1. NMR spectroscopy

$^{1}$H NMR spectrum of TCAD was shown in Figure S5. There is only one signal associated with the compound (8.56 ppm). It is very wide because amine protons are acidic and they undergo exchange.

In the $^{13}$C NMR spectrum (Figure S6) there are three signals which come from TCAD carbons (112.9, 119.2, 159.7 ppm). The narrow range was presented in the Figure S7 to show that 159.7 ppm signal is a doublet.

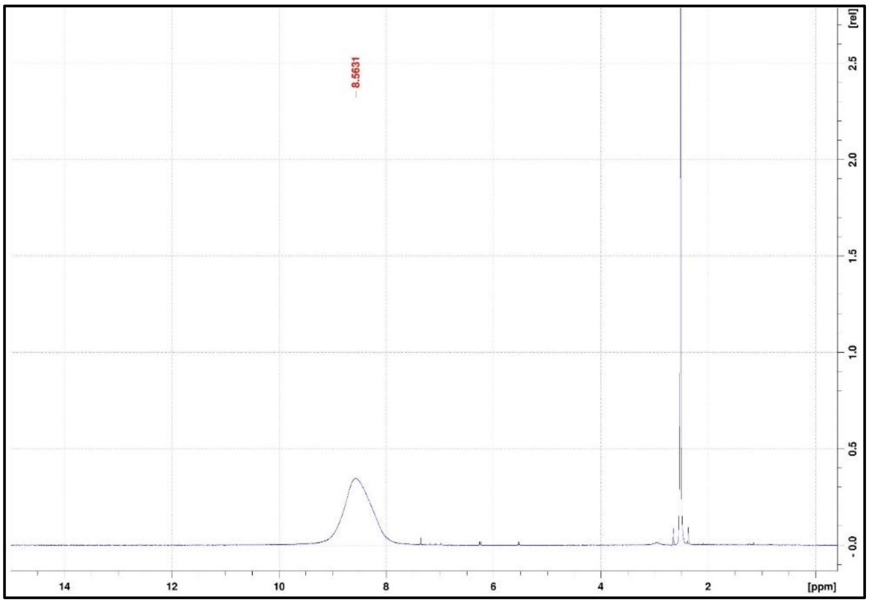


**Figure S5** $^{1}$H NMR spectrum of **TCAD.**

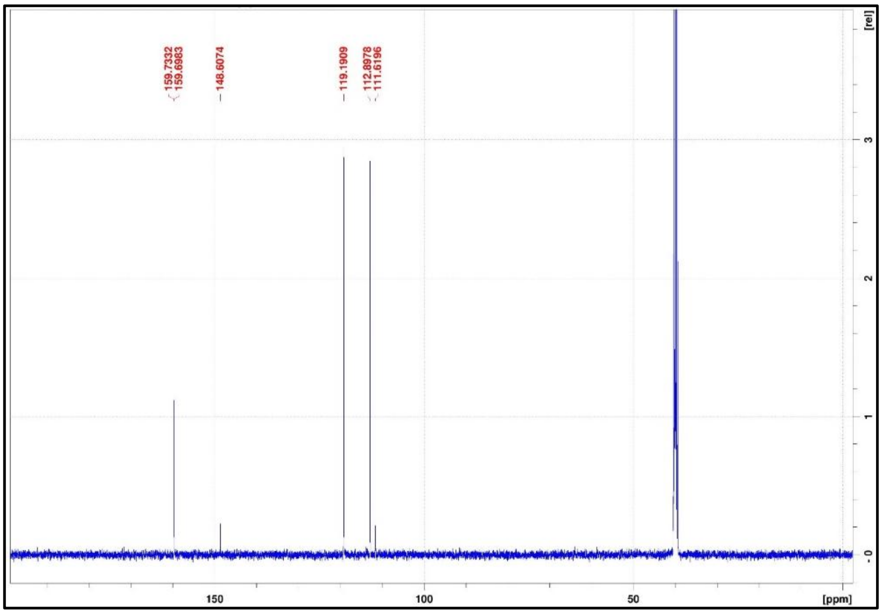


**Figure S6.** $^{13}$C NMR spectrum of **TCAD**.

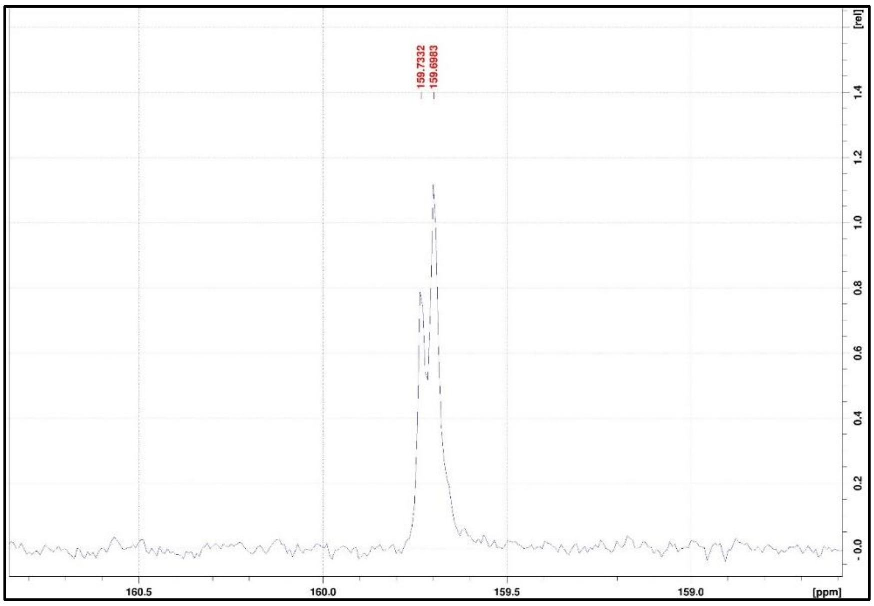


**Figure S7.** $^{13}C$ NMR spectrum of **TCAD** in the 158.5- 161.0 ppm range.

### 3.2. FTIR spectroscopy

FTIR spectrum of **TCAD** was shown in Figure S8. Some characteristic bands of absorption were observed [$cm^{-1}$]: 3162 (N-H, stretch.), 2247 (nitrile group, stretch.), 1540 and 1421 (imidazole ring, stretch.), 1421 (N=N, stretch.)

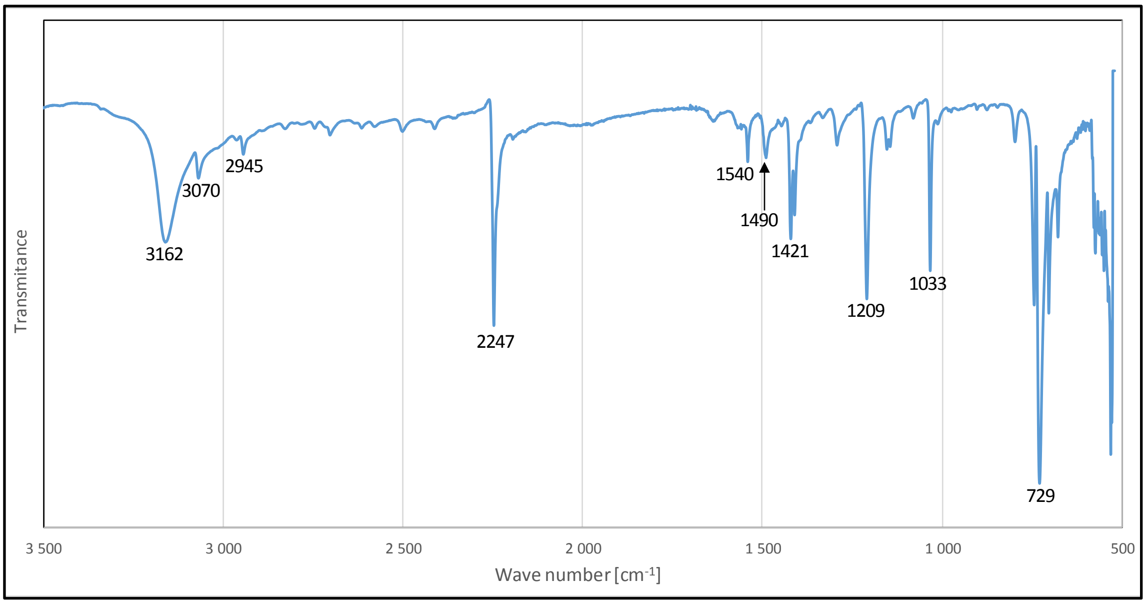


**Figure S8.** FT-IR spectrum of **TCAD**.

## 4. Thermal analysis

DTA/TG curves of **TCAD** with a heating rate of 5 °C/min were shown in Figure S9. The thermogram was described in the main article. In the next pictures there are

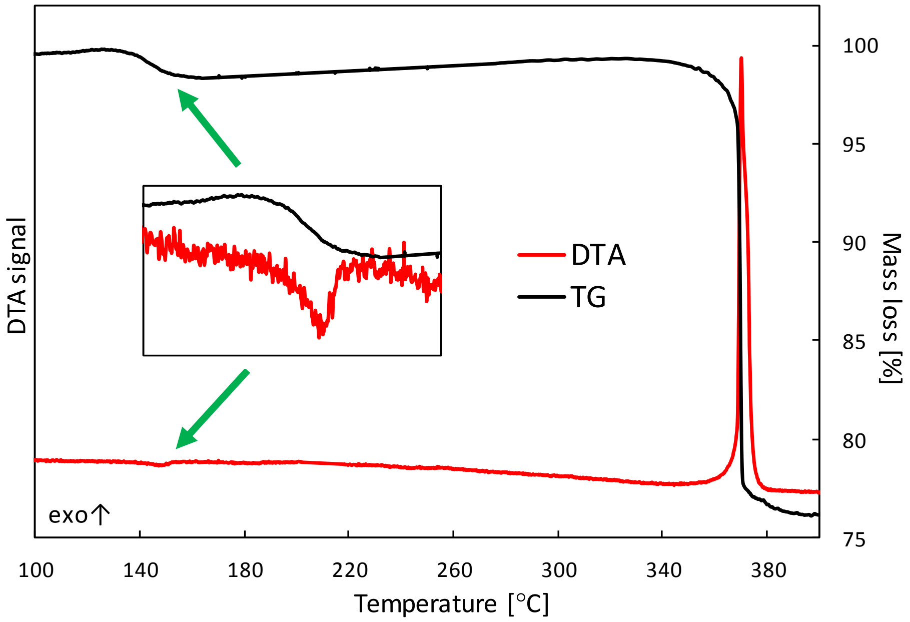


DTA (Figure S10) and TG (Figure S11) curves registered for different heating rates.

**Figure S9.** DTA/TG thermogram of **TCAD** (heating rate: 5 °C/min).

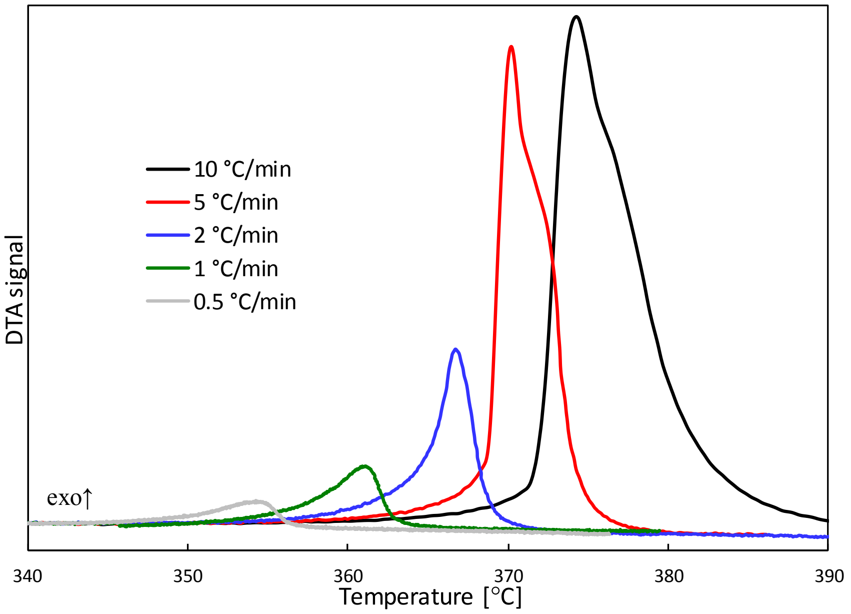


**Figure S10.** DTA curves of **TCAD** for various heating rates.

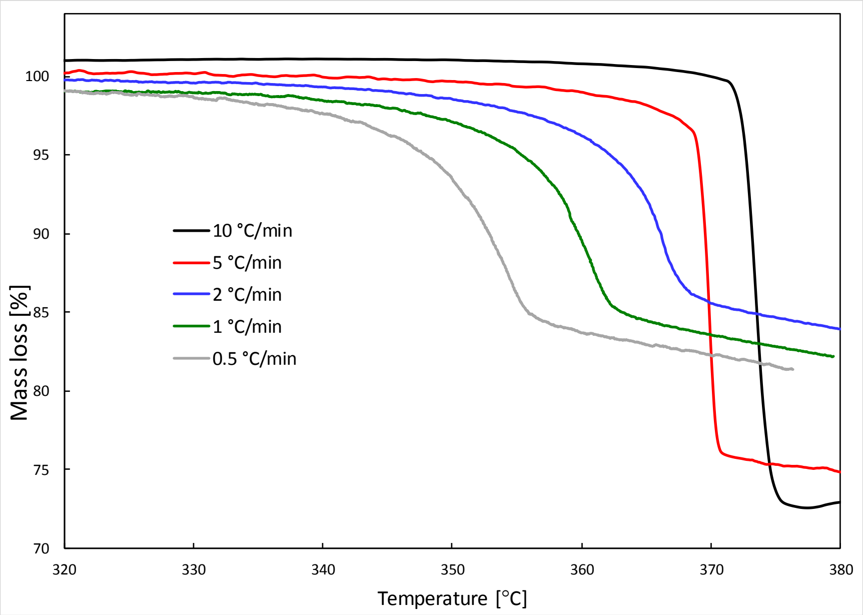


**Figure S11.** TG curves of **TCAD** for various heating rates.

# References


1. Spackman, M.A.; Jayatilaka, D. Hirshfeld surface analysis. *CrystEngComm* **2009**, *11*, 19-32, DOI:10.1039/B818330A.
2. Wolff, S.K.; Grimwood, D.J.; McKinnon, J.J.; Turner, M.J.; Jayatilaka, D.; Spackman, M.A. *CrystalExplorer (Version 3.1)*; University of Western Australia, 2012.
3. Frisch, M.J.; Trucks, G.W.; Schlegel, H.B.; Scuseria, G.E.; Robb, M.A.; Cheeseman, J.R.; Scalmani, G.; Barone, V.; Mennucci, B.; Petersson, G.A., et al. *Gaussian 09, Revision E.01*, Gaussian, Inc., Wallingford CT, 2009.
4. Montgomery, J.A.; Frisch, M.J.; Ochterski, J.W.; Petersson, G.A. A complete basis set model chemistry. VII. Use of the minimum population localization method. *J. Chem. Phys.* **2000**, *112*, 6532-6542, DOI:10.1063/1.481224.
5. Ochterski, J.W.; Petersson, G.A.; Montgomery, J.A. A complete basis set model chemistry. V. Extensions to six or more heavy atoms. *J. Chem. Phys.* **1996**, *104*, 2598-2619, DOI:10.1063/1.470985.
6. Byrd, E.F.C.; Rice, B.M. Improved prediction of heats of formation of energetic materials using quantum mechanical calculations. *J. Phys. Chem. A* **2006**, *110*, 1005-1013, DOI:10.1021/jp0536192.
7. Rice, B.M.; Pai, S.V.; Hare, J. Predicting heats of formation of energetic materials using quantum mechanical calculations. *Combust. Flame* **1999**, *118*, 445-458, DOI:10.1016/S0010-2180(99)00008-5.
8. Curtiss, L.A. Assessment of Gaussian-2 and density functional theories for the computation of enthalpies of formation. *J. Chem. Phys.* **1997**, *106*, 1063-1079, DOI:10.1063/1.473182.
9. Linstrom, P.J.; Mallard, W.G. *NIST Chemistry WebBook, NIST Standard Reference Database Number 69*; National Institute of Standards and Technology: Gaithersburg, MD 20899, 2017.
10. Trouton, F. On molecular latent heat. *Philos. Mag.* **1884**, *18*, 54-57, DOI:10.1080/14786448408627563.
11. Rice, B.M.; Hare, J.J. A quantum mechanical investigation of the relation between impact sensitivity and the charge distribution in energetic molecules. *J. Phys. Chem. A* **2002**, *106*, 1770-1783, DOI:10.1021/jp012602q.
12. Dennington, R.; Keith, T.; Millam, J. *GaussView, Version 5; Semichem*; Shawnee Mission, KS, USA, 2009.
